# Short Course for Focused Assessment with Sonography for Human Immunodeficiency Virus/Tuberculosis: Preliminary Results in a Rural Setting in South Africa with High Prevalence of Human Immunodeficiency Virus and Tuberculosis

**DOI:** 10.4269/ajtmh.2010.09-0561

**Published:** 2010-03

**Authors:** Tom Heller, Claudia Wallrauch, Richard J. Lessells, Sam Goblirsch, Enrico Brunetti

**Affiliations:** Hlabisa Hospital, Hlabisa, KwaZulu-Natal, South Africa; Africa Centre for Health and Population Studies, University of KwaZulu-Natal, Somkhele, KwaZulu-Natal, South Africa; Department of Medicine-Pediatrics, University of Minnesota, Minneapolis, Minnesota; Division of Infectious and Tropical Diseases, University of Pavia, Istituto Di Ricovero e Cura a Carattere Scientifico San Matteo Hospital Foundation, Pavia, Italy

## Abstract

In Africa, human immunodeficiency virus (HIV)–associated extrapulmonary tuberculosis (TB) is common and poses diagnostic difficulties. Ultrasound is useful to find suggestive signs such as effusions or abdominal lymphadenopathy. Because trained radiologists are scarce in resource-poor settings, even this simple and relatively inexpensive diagnostic tool is frequently unavailable to patients in district hospitals in sub-Saharan Africa. We developed a focused protocol for assessment with sonography for HIV/TB and trained physicians in a rural district hospital in South Africa. In this pilot study, high levels of confidence in identifying specific signs were rapidly achieved and ultrasound was introduced into routine clinical practice.

In sub-Saharan Africa, the convergence of human immunodeficiency virus (HIV) and tuberculosis (TB) epidemics has led to a resurgence of extrapulmonary TB (EPTB), such that in 2007 EPTB accounted for 19% of all TB cases reported by directly observed treatment, short-course programs in the African region.^1^ Although pulmonary TB remains the focus of TB control efforts, the morbidity and mortality associated with extrapulmonary TB, especially in association with HIV infection, warrant increased attention.^2^ Common manifestations of EPTB include pleural effusion, pericardial effusion, and abdominal TB. Diagnosis is hampered by the poor sensitivity of microscopy and the limited availability of culture techniques; consequently the diagnosis is usually based on clinical case definitions.^3^

Ultrasound has the potential to aid TB diagnosis by rapidly identifying abnormal signs, which in high prevalence settings will be highly suggestive of EPTB.^4^ Because trained ultrasonographers are scarce in rural, limited-resource settings, short training courses for physicians (or other health care workers) on ultrasound, focused on limited key findings, may be an option to achieve wider coverage. The potential public health benefit of this in rural Africa is far reaching, in terms of more rapid identification of EPTB cases at a peripheral clinic level, where most HIV care will be delivered in the future. In emergency medicine, short protocols such as focused assessment with sonography for trauma (FAST) have been successfully established for more than a decade.^5^,^6^

Hlabisa Hospital is a 300-bed district hospital in northern KwaZulu-Natal, South Africa. The HIV prevalence in the adult resident population (age range = 15–49 years) is 21.5%.^7^ The TB notification rate in 2008 was approximately 1,700 cases per 100,000 person-years (Department of Health, Umkhanyakude District, unpublished data), and 76% of TB cases are co-infected with HIV.

A focused assessment with sonography for HIV/TB (FASH) protocol was developed by one of the authors (T.H.), an internal medicine physician with more than 10 years of experience in medical ultrasound. Modeled on the FAST protocol and experiences from the field of emergency medicine, a list of key ultrasound findings were identified and included in the protocol. The selection criteria were based on the relative ease to recognize the finding and the relevance with respect to diagnostic and therapeutic decisions. Pericardial, pleural, and abdominal effusions (ascites) were identified as FASH findings. The FASH-plus findings, which were considered more difficult to recognize, were upper abdominal lymph nodes (> 1.5 cm in diameter), focal splenic lesions, and focal liver lesions. A pictorial summary of the protocol is displayed in Figure 1.

**Figure 1.**
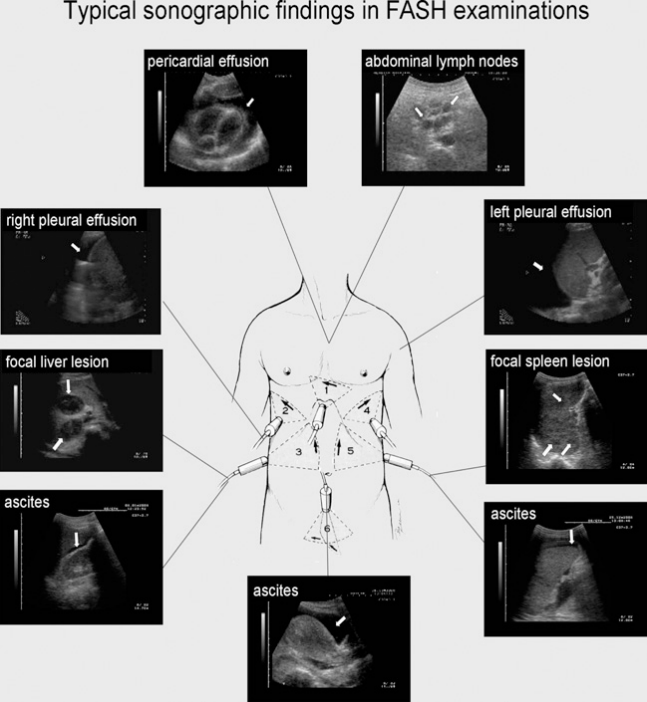
Pictorial summary of the focused assessment with sonography for human immunodeficiency virus/tuberculosis (HIV/TB) (FASH).

The training course was conducted over two consecutive days for 3–5 participants in each sitting. All participants were trained in general medicine but had no previous experience of ultrasonography or radiology. Approximately 8 hours of theoretical lectures were presented including video clips of relevant pathologies. Afternoon sessions included applied practical training initially using course participants, as models for normal anatomy, and then with hospital inpatients for a combined total of 6–8 hours. The curriculum of the course is summarized in Table 1. Two conventional black and white ultrasound scanners were used (Just-Vision 400-Model SSA-325A; Toshiba, Tokyo, Japan and Merlin, BK Medical, Copenhagen, Demark), both with 3.5-MHz abdominal probes.

Three participants in the first course subsequently documented their first approximately 20 examinations on a standardized report form to evaluate the effect of the training. Only HIV-positive inpatients were included. Subjective confidence with the technique was rated on a scale of 1 to 5 (1 = highly confident, 5 = not confident). The duration of the examination and the requirement for a second opinion were documented. All ultrasound examinations were performed without direct supervision as part of routine medical care.

After these 20 preliminary examinations, the three participants performed five study examinations each and recorded the results. All patients were re-examined on the same day by the reference examiner (T.H.) who was blinded to the results of the participants. For each examination, the presence or absence of the six key sonographic findings was recorded; thus, 90 pairs of findings were compared. To assess the inter-observer agreement the kappa test ratio (κ) was calculated according to the equation κ = (Observed agreement – chance agreement)/1 – chance agreement. A κ value > 0.8 is usually accepted as a sign of very good agreement.^8^ Using the reference examiner as the gold standard, we calculated the sensitivity and specificity of the participants' ultrasound examinations.

Three junior hospital physicians participated in the study and performed ultrasound examinations after the initial FASH course and recorded their results. A total of 62 examinations were analyzed. All patients examined were HIV positive; median CD4 cell count was 128 cells/mm^3^. The most common indications for ultrasound were abdominal pain (often with concurrent fever or abnormal liver function test results) in 25 patients (40%), cardiomegaly on chest radiograph in 10 patients (16%), fever of unknown origin in 8 patients (13%), and suspected pleural effusion on chest radiograph in 5 patients (8%).

The participants reported their findings on a standardized form. Effusions were found in a high proportion of the examinations: pericardial effusion in 32 examinations (52%), ascites in 14 (23%), and pleural effusion in 13 (21%). Abdominal lymph nodes (> 1.5 cm in diameter) were seen in 18 examinations (29%), focal splenic lesions in 10 (16%), and focal liver lesions in 2 (3%).

The mean time spent per examination was 7.2 minutes. In more than 84% of the examinations, the participants were subjectively confident or highly confident in being able to identify or exclude free fluid. The level of confidence with respect to the FASH-plus findings was lower: in 61% of the examinations, the participants were highly confident or confident with their findings. During their first five examinations, each participant requested a second opinion to verify their results in 60% of the examinations; this rate decreased to 23% in subsequent examinations.

In 29 of 62 patients (47%), patient management was changed as a result of the FASH examination (TB treatment was given to 15 patients; steroids were also given for TB pericarditis to 10; furosemide with or without enalapril was given to 4 because cardiomyopathy rather than pericardial effusion was identified as a cause of cardiomegaly). In 7 patients with CD4 cell counts > 200 cells/mm^3^, antiretroviral therapy was given because extrapulmonary TB (World Health Organization stage IV condition) qualified patients for antiretroviral treatment according to South African guidelines.

Fifteen study examinations (five for each participant) were performed after the training to determine the concordance between the participants and the reference examiner. The key findings were identified as follows by the reference examiner: pericardial effusion (3 examinations), pleural effusion (4), ascites (4), abdominal lymph nodes (7), and focal splenic lesion (4). None of the examinations included focal liver lesions. There were two instances of discrepant findings: abdominal lymph nodes were not detected by the participant in one patient; and pericardial effusion was reported by the participant but not by the reference examiner in one patient. The κ test yielded a result of 0.94 (95% confidence interval [CI] = 0.85–1.00), which indicated a very good agreement between the observers. Using the reference examiner as a gold standard, we determined that the participants' examinations had a sensitivity of 95.5% (95% CI = 78.2–99.1) and specificity of 98.5% (95% CI = 92.1–99.7) to identify the sonographic findings.

The diagnosis of extrapulmonary TB in HIV patients is challenging, especially in the resource-poor setting. The potential exists to improve the diagnostic pathways for suspected extrapulmonary TB by integrating the standardized ultrasound protocol described in this report. Pleural effusion, especially when unilateral and associated with HIV infection, is most likely to be caused by tuberculosis in countries with high TB incidence.^9^ Tuberculosis is also consistently reported as the predominant cause of pericardial effusion and in most cases is associated with HIV co-infection.^10^ Enlarged lymph nodes and splenic micro-abscesses are not included in current World Health Organization guidelines, but have been described as characteristic findings by our own group^11^ and in other HIV-positive patient populations.^4^ It might prove useful to add these to future diagnostic algorithms because they are findings highly suggestive of abdominal TB in HIV patients.

Diagnostic ultrasound is feasible in the rural health care setting in African countries and can improve case management in many clinical problems.^12^,^13^ It has to be noted that ultrasound is a highly operator-dependent tool. The World Health Organization scientific Group on Clinical Diagnostic Imaging concluded that “…more important than the equipment is the availability of skills.” The report further stated that “worldwide, it is likely that much of the ultrasonography currently performed is carried out by individuals with in fact little or no formal training.”^14^ Despite this fact, reports on ultrasound training programs in district hospitals are scarce and programs are often too long and time consuming to achieve wider coverage.^15^ Shorter and focused ultrasound courses may pose an alternative approach to increase the availability and utility of ultrasound at least for the diagnosis of the locally most prevalent and important conditions.

Our study can be seen as a proof of concept of the course, although there are some study limitations: the number of participants evaluating the course is low and further assessment in larger groups of participants and in different patient populations should follow. To assess the accuracy of the participants' observations, these observations were compared with the instructor's examinations as a reference. This comparison can at best serve as an imperfect gold standard; thus, the accuracy might be overestimated.^16^ Nevertheless, the high concordance can be seen as a promising result. It further has to be understood that although we describe the accuracy of detecting ultrasound findings suggestive of TB in HIV patients, the final diagnosis of the disease must be based on additional clinical and/or laboratory findings.

Focused assessment with sonography for HIV/TB is a technique that can be taught rapidly to physicians with little or no prior ultrasound experience. The learning process is facilitated by the fact that pathologic findings are present in a large proportion of hospital inpatients. Persons therefore rapidly gain confidence in the identification of key pathological findings. The fact that many patients under investigation are underweight makes scanning easier because interfering fat and colon gas become less relevant. The examination takes only a few minutes and may provide important findings that alter patient management. Further work is required to establish whether these findings can be replicated in other settings, particularly where the pre-test probability of significant pathologic changes is lower. Of particular interest would be the opportunity to implement a similar training course at the primary health care level given that this is where most HIV care in Southern Africa will be delivered in the future.

## Figures and Tables

**Table 1:** Curriculum of the focused assessment with sonography for human immunodeficiency virus/tuberculosis training*

PHYSICS AND INSTRUMENTATION A. General Physics 1. Frequency 2. Wave length 3. Speed 4. Piezoelectric effect 5. Attenuation 6. Tissue Interaction a. Reflection b. Transmission 7. Safety B. Instrumentation 1. Probe Types a. Linear array b. Phased array 2. Receiver Controls a. Gain b. Compensation (TGC) C. Imaging Artefacts 1. Reverberation 2. Refraction 3. Mirror image 4. Shadowing 5. Enhancement
Educational Methods 1. Lecture [1 hour]
CARDIAC A. Anatomy 1. Cardiac Chambers 2. Myocardium 3. Pericardium B. Techniques 1. Standard Windows C. Pericardial Fluid 1. Definition 2. Etiologies 3. Sonographic features D. Cardiac Tamponade 1. Definition 2. Sonographic features 3. Interventional treatment
Educational Methods 1. Lecture [1 hour] 2. Case Reviews [1/2 hour]
THORACIC A. Anatomy 1. Chest wall 2. Pleural cavity 3. Lung B. Techniques 1. Standard Window C. Pleural Fluid 1. Definition 2. Etiologies 3. Sonographic features 4. Treatment
Educational Methods 1. Lecture [1/2 hour] 2. Case Reviews [1/2 hour]
ABDOMINAL A. Anatomy 1. Liver 2. Diaphragm 3. Spleen 4. Kidneys 5. Pancreas 6. Bowel B. Techniques 1. Standard Windows 2. Orientation C. Abdominal Fluid 1. Fluid-Locations a. Hepatorenal recess (Morrison's pouch) b. Splenorenal recess c. Douglas' pouch d. Subdiaphragmatic D. Liver abnormalities a. Intrahepatic abscesses b. Other focal findings c. Cirrhosis E. Lymph nodes a. Celiac/portal lymph nodes b. Paraaortal lymph nodes c. Caecal lymph nodes d. Superficial lymph nodes F. Spleen a. Splenomegaly b. Splenic microabcesses
Educational Methods 1. Lecture [3 hour] 2. Case Reviews [1 hour]
INTERVENTIONAL ULTRASOUND A. Procedures-Ultrasound Guided 1. Pericardiocentesis 2. Thoracentesis 3. Paracentesis
Educational Methods 1. Lecture [1 hour]
TECHNICAL SKILLS
Educational Methods 1. Practical training [6 hours]

* TGC = time gain compensation.
